# Rapid defense mechanism suppression during viral- oomycete disease complex formation

**DOI:** 10.3389/fpls.2023.1124911

**Published:** 2023-06-09

**Authors:** Amit M. Philosoph, Aviv Dombrovsky, Neta Luria, Noa Sela, Yigal Elad, Omer Frenkel

**Affiliations:** ^1^ Department of Plant Pathology and Weed Science, The Volcani Institute, Agricultural Research Organization, Bet Dagan, Israel; ^2^ The Robert H. Smith Faculty of Agriculture, Food and Environment, The Levi Eshkol School of Agriculture, The Hebrew University of Jerusalem, Rehovot, Israel

**Keywords:** below ground above ground interactions, combined infections, cross talk, pathobiome, Pythium, soil borne pathogens, tobamovirus

## Abstract

Combined infection of the host plant with pathogens involving different parasitic lifestyles may result in synergistic effects that intensify disease symptoms. Understanding the molecular dynamics during concurrent infection provides essential insight into the host response. The transcriptomic pattern of cucumber plants infected with a necrotrophic pathogen, *Pythium spinosum*, and a biotrophic pathogen, Cucumber green mottle mosaic virus (CGMMV) was studied at different time points, under regimes of single and co-infection. Analysis of CGMMV infection alone revealed a mild influence on host gene expression at the stem base, while the infection by *P. spinosum* is associated with drastic changes in gene expression. Comparing *P. spinosum* as a single infecting pathogen with a later co-infection by CGMMV revealed a rapid host response as early as 24 hours post-CGMMV inoculation with a sharp downregulation of genes related to the host defense mechanism against the necrotrophic pathogen. Suppression of the defense mechanism of co-infected plants was followed by severe stress, including 30% plants mortality and an increase of the *P. spinosum* hyphae. The first evidence of defense recovery against the necrotrophic pathogen only occurred 13 days post-viral infection. These results support the hypothesis that the viral infection of the Pythium pre-infected plants subverted the host defense system and changed the equilibrium obtained with *P. spinosum*. It also implies a time window in which the plants are most susceptible to *P. spinosum* after CGMMV infection.

## 1 Introduction

During plant development through the growing season, plants may encounter multiple pathogen attacks, including co-infection with different pathogens and lifestyles (Abdullah et al., 2017). Infections can include pathogen complexes of the same kingdom or cross-kingdom (Lamichhane and Venturi, 2015). Co-infection may cause diverse epidemiological and phenotypic outcomes that influence plant survival, biomass or seed production, substantially differing from infection by a single pathogen (Tollenaere et al., 2016; Tang et al., 2019).

A major pitfall of studying the co-infection process is the paradigm that most co-infection studies occur simultaneously; however, often there is a temporal gap between the infection of the pathogens, and the first pathogen may already have reached an equilibrium with the host (Chávez-Calvillo et al., 2016; Karvonen et al., 2019). Under these circumstances, in an antagonistic interaction scenario, the first colonizer suppresses the second, and the effect of the second pathogen of the disease complex is reduced (Chávez-Calvillo et al., 2016; Verbeek et al., 2019). In the second scenario, the first infection is constrained by the host plant, but the second pathogen’s introduction destabilizes the plant defenses, causing synergistic damage. The apparent damage might be substantially different from the damage caused by each pathogen alone, such as higher disease severity or even mortality (Spoel et al., 2007; Tang et al., 2019).

One exciting scenario is that co-infection of pathogens with different lifestyles may initiate a cross-reactive immune response (Spoel et al., 2007). Systemic immunity can be divided into systemic acquired resistance (SAR) and induced systemic resistance (ISR), depending on induction site and the lifestyle of the inducing microorganism (Vlot et al., 2021). In general, biotrophic pathogens including viruses, and some fungi and bacteria promote systemic acquired resistance (SAR) that involves the synthesis of pathogenesis-related proteins, phytoalexins and the induction of hypersensitive responses (HR), often related to programmed cell death, which restrict pathogen colonization in the infected area (Conrath, 2006). These responses are typically mediated by the phytohormone salicylic acid (SA) (Klessig et al., 2018). In contrast, induced systemic resistance (ISR) that induced by beneficial microbes as well as necrotrophic pathogens, enhance the phytohormones jasmonic acid (JA) and/or ethylene (ET) (Vlot et al., 2021). In addition to JA role in regulating plant defense responses against biotic stress, it is also required for plant reproduction and other growth and developmental processes (Huang et al., 2017).

To date, the cross communications of plant defense pathways were studied at different regulatory layers, including gene expression and phytohormone metabolism (Koornneef and Pieterse, 2008; Li et al., 2019). Growing evidence supports the fact that JA, SA, and ET defense signaling pathways are involved in a multifaceted signaling network in which the different pathways affect each other through negative and positive regulatory interactions (Robert-Seilaniantz et al., 2011). Although cross-communication between the hormone signaling pathways can cause synergistic interactions (Mur et al., 2006), most studies indicate a mutually antagonistic interaction between SA- and JA-dependent signaling (Kunkel and Brooks, 2002; Beckers and Spoel, 2006). As a result of this negative cross-talk between SA and JA, activation of the SA response by a biotrophic pathogen may lead to higher susceptibility of plant tissues to infection by necrotrophic pathogen by suppressing the JA-signaling pathway (Spoel et al., 2007; Caarls et al., 2015). Nevertheless, the complex interactions between many hormone signaling pathways may include additional pathways to suppress the ISR response (Zhang et al., 2018; Yang et al., 2019).

The outcome of combined infection with necrotrophic and biotrophic pathogens is also highly relevant in agricultural and natural pathosystems (Tang et al., 2019; Karvonen et al., 2019), directly affecting disease epidemics and crop production (Lamichhane and Venturi, 2015). A recent study of an emerging phenomenon that causes extensive collapse of cucumber plants in greenhouses, that until recently was attributed solely to Cucumber green mottle mosaic virus (CGMMV), revealed the crucial involvement of *Pythium spinosum* in this wilting syndrome. (Philosoph et al., 2018; Philosoph et al., 2019).

The tobamovirus CGMMV, with a single-stranded positive-sense RNA genome, was first reported in 1935 in cucumber (*Cucumis sativus L*.) (Adams et al., 2009). In the last 15 years CGMMV has become a significant threat in more than 30 growing countries for melon (*Cucumis melo*), watermelon (*Citrullus lanatus*) and cucumber (Sun et al., 2019). The main symptoms of CGMMV include mottle and mosaic patterning of infected leaves, distorted fruit and reduced yield (Park et al., 2005; Dombrovsky et al., 2017). CGMMV is easily spread by mechanical contact, and seeds (Reingold et al., 2016). Observations in commercial trellised cucumber greenhouses that documented CGMMV spread revealed extensive collapse and growth inhibition of plants 3 to 6 weeks post-planting (Antignus et al., 2001; Ayo-John et al., 2014) due to combined infection of CGMMV with the soilborne pathogen *Pythium spinosum* (Philosoph et al., 2018). This oomycete pathogen infects several hosts, including cucumbers (Al-Sa'di et al., 2007; Toporek and Keinath, 2020) resulting in damping-off and root rot in very young seedlings, but rarely causes apparent damage in mature plants (Hendricks and Roberts, 2015; Sigillo et al., 2020). Our recent work showed that the combined infection of *P. spinosum* with CGMMV leads to an extensive late wilting disease and growth constraint of mature cucumber plants (**Figure S1**). The disease was apparent in a range of environmental conditions and was synergistic, regardless of the interval between infection by the oomycete and the virus (Philosoph et al., 2018; Philosoph et al., 2019). In addition, we showed that the wilting syndrome starts several days before the appearance of the viral symptoms (Philosoph et al., 2019), raising the hypothesis that the viral infection of the Pythium pre-infected plants subverted the host defense system and changed the equilibrium obtained with Pythium. This pathosystem provides a valuable case study to increase our understanding of the temporal changes during the co-infection process, with CGMMV acting as a biotroph and *P. spinosum* as a necrotroph. The research goal was to delve into the processes involved in the cucumber plant collapse during co-infection of *Pythium* and CGMMV by conducting an in depth molecular characterization. Hence, we performed transcriptomic analysis to illuminate the major gene expression changes that occurred in the plants infected with *Pythium* prior to CGMMV inoculation, and subsequently at different times after the infection of CGMMV in healthy or Pythium-infected plants.

## 2 Material and methods

### 2.1 Plant growing conditions

Cucumber (*C. sativus* cv. Kfir, Zeraim Gedera, Israel) seedlings were grown in a nursery tray with a potting mixture (Even-ARI, Israel) in controlled environment growth chambers at 22 ± 1°C. Seedlings were kept under a 12 h photoperiod and were fertilized and drip-irrigated twice a day (~0.1 l per irrigation) with 5:3:8 NPK fertilizer (N-120 mg/l; P-30 mg/l; and K-50 mg/l).

### 2.2 Pathogen growth and inoculation

*Pythium spinosum* (PS-01 isolate, GenBank accession number MF116303) was cultured on PDA (Difco Laboratories) and incubated at 25°C for 2 days. Six agar disks (9 mm diameter) were cut from the periphery of the colony and placed in a 500 ml Erlenmeyer containing 80 g autoclaved pearl millet. The *P. spinosum*-colonized millet was incubated for 6 days at 22°C ± 1°C and then homogenized, adjusted to 0.25% (w/w) with Vermiculite (Agrekal, Israel). The control treatment contained a mix of 0.25% non-colonized millet seeds. CGMMV inoculation was performed as previously described (Reingold et al., 2015). Briefly, cotyledons of cucumber plants were gently rubbed with phosphate buffer (0.01 M, pH 7) containing carborundum dust (silicon carbide) and extract of CGMMV-inoculated cucumber leaves (Ah isolate, GenBank accession number KF155232). Non-inoculated plants and plants infected only with *P. spinosum* were similarly treated with virus-free buffer and carborundum.

### 2.3 Controlled-environment experiments and sample collection

Six days after sowing, 300 seedlings were transplanted into *P. spinosum*-inoculated vermiculite medium, and an additional 100 seedlings were transplanted into non-inoculated control medium. Each cucumber seedling was transplanted to a 100 ml pot (Kolbolagan, Israel) containing 100 g vermiculite and grown in greenhouse conditions described above. Five days after the *P. spinosum* inoculation, which permitted observation of the primary “damping off” of seedlings due to *Pythium* alone (Philosoph et al., 2018), 100 symptomless plants were taken to the core experiment, along with the 100 non-inoculated plants. Fifty plants from each group were then inoculated with CGMMV as described above, forming four different treatments: (i) plants infected with *P. spinosum* inoculated with CGMMV (PS+CG); (ii) plants with *P. spinosum* (PS); (iii) plants inoculated solely with CGMMV (CG), and (iv) non-inoculated healthy control plants (C).

The first collection of samples began on the same day as the CGMMV inoculation (T0); samples were taken from the collar-region (where the hypocotyl meets the root) of 5 cucumber plants inoculated with *P. spinosum* and 5 non-inoculated control plants to characterize the plant response before the CGMMV inoculation. Subsequently, the samples were taken from the collar-region of all the four treatments described above (5 plants per treatment) 1, 2, 3, 6 and 13 days post-viral inoculation (dpvi) with CGMMV. All samples were collected in the controlled-environment chamber directly into a test tube (Eppendorf Tubes^®^, Hamburg, Germany) with liquid nitrogen and stored at -80°C for later RNA extraction. In addition, the roots of all plants were tested for the presence of *P. spinosum* using selective corn meal agar, as described by Philosoph et al. (2018).

### 2.4 RNA sequencing analysis

At the end of the experiment, total RNA was extracted from all cucumbers’ collar-region samples (~200 mg) using the GenElute mammalian total RNA miniprep kit (Sigma-Aldrich, USA). Firstly, samples were placed in lysis buffer and mercaptoethanol with two 5-mm-diameter tungsten balls, and tissue was grounded using FastPrep-24 5G Instrument (MP Biomedicals, Santa Ana, California) at 6 m/s for 40 s for two cycles. The extraction proceeded according to the manufacturer’s protocol. Extracted RNA was treated with DNase (TURBO DNA-free Kit, Ambion Life Technologies, USA). RNA yield and purity were measured by Nanodrop (ND-1000 Spectrophotometer, Wilmington, USA) and validated for quality by running an aliquot on a Bioanalyzer 2200 TapeStation (Agilent Technologies, California). For each treatment, at each time point, libraries were prepared from up to four biological replicates. Single-end RNA-seq libraries (TruSeq RNA Library Prep Kit v2) (50 bp) were prepared and sequenced in the Technion Institute of Technology on an Illumina HiSeq 2500 machine. RNAseq raw data reads are publicly available in the NCBI under BioProject ID PRJNA808669. Raw reads (FastQ files) were inspected for quality with FastQC v0.11.5 (https://www.bioinformatics.babraham.ac.uk/projects/fastqc/) and trimmed for quality and adaptor removal using Trim Galore default settings. (https://github.com/FelixKrueger/TrimGalore). The average raw reads library size per sample was ~26.9 M. An average of 1.28% of the reads were trimmed. Trimmed reads were mapped to Cucumber_ChineseLong_v2 genome as downloaded from http://cucurbitgenomics.org/ftp/genome/cucumber/Chinese_long/v2/by using STAR mapper v. 2.6.0c (Dobin et al., 2013). RSEM package (Li and Dewey, 2011) was used for quantifying of genes and isoform abundances. The average percent of alignable reads was 68% from which, an average of 97% were aligned uniquely. Expected counts of mapped reads both to genes and transcripts of all samples were pooled and subjected to differential expression analysis using the DESeq2 R package (Love et al., 2014). Principal Component Analysis (PCA) were generated using R precomp function with several different subsets of samples to reduce the experiment design’s complexity.

### 2.5 Differential expression and gene set enrichment analyses

The differential expression analysis of genes was determined based on the read counts of expressed genes using DESeq2 package in R with a significance of False Discovery Rate (FDR ≤ 0.05) (Love et al., 2014). The threshold of log2 fold change greater then > 2 or smaller than < -2, and *P*-adjusted (FDR) values below 0.05 was used to identify Differentially Expressed Genes (DEGs) for each treatment comparison.

To characterize the DEGs of plants infected with *P. spinosum* just before the inoculation with CGMMV, a comparison between *P. spinosum* and control at T0 was performed. For Differentially expressed genes annotation we used blastx within the Galaxy platform (https://usegalaxy.org) (Cock et al., 2015). Then we did functional classification and prediction by using Clusters of Orthologous Groups (COG) analysis (Tatusov et al., 2003) and eggnog-mapper (Huerta-Cepas et al., 2017), based on eggNOG v4.5 orthology data (Huerta-Cepas et al., 2016). Gene Ontology (GO) enrichment analysis for the DEGs was performed by AmiGO 2 *via* Cucurbit Genomics Database (CuGenDB-http://cucurbitgenomics.org/) with a threshold of FDR <0.05. The REVIGO program (http://revigo.irb.hr/) was used for visualization of enriched GO biological process terms (Supek et al., 2011) and the interaction between GO biological process was visualized by using Cytoscape V3.8.0 (Shannon et al., 2003). Pathways enrichment analysis of upregulated and downregulated genes was performed using Kyoto Encyclopedia of Genes and Genomes (KEGG) mapper (http://www.genome.jp/kegg/mapper.html) and CuGenDB (FDR<0.05).

### 2.6 Characterization of plants inoculated with CGMMV

To study the influence of CGMMV inoculation, a comparison was conducted between plants infected with CGMMV versus (vs) healthy control plants at each time point, starting from 24 hours after inoculation (D1) and in subsequent days (2, 3, 6, and 13 dpvi). The DEGs obtained from these comparison pathways were enriched using KEGG mapper and CuGenDB (FDR < 0.05).

The next step was to test the CGMMV inoculation effect on plants previously infected with *P. spinosum*, therefore comparing co-infected plants inoculated with *P. spinosum* and CGMMV (PS+CG) vs plants infected with *P. spinosum*. KEGG was used to predict the DEGs’ enriched pathways by using KOBAS to test the statistical enrichment of DEGs in KEGG pathways (Xie et al., 2011), and data were visualized in scatterplot by using Plotly (https://plotly.com/python). The DEGs data were adjusted through quantile normalization and then standardized using EXpression Analyzer and DisplayER (EXPANDER) v7.0 (Shamir et al., 2005). After normalization, the average signal value of the biological replicates for each sample was used to perform hierarchical clustering analysis and Heatmap using the ClustVis tool (https://biit.cs.ut.ee/clustvis/) (Metsalu and Vilo, 2015). Pathways enrichment analysis of upregulated and downregulated genes was performed using KEGG mapper (Mao et al., 2005) and CuGenDB (FDR<0.05). The overlapping downregulated DEGs in each pathway from “PS+CG vs control”, and upregulated DEGs in each pathway from “*P. spinosum* vs control” analysis, were determined by Venny 2.1 (https://bioinfogp.cnb.csic.es/tools/venny/index.html).

### 2.7 Gene expression analysis by real-time quantitative RT-PCR

Total RNA was reverse‐transcribed using Verso cDNA Synthesis Kit (Thermo Scientific™, USA). Relative quantification of selected mRNA defense-related genes was performed (Livak and Schmittgen, 2001). Primers for all tested genes were designed using Primer3plus (http://www.bioinformatics.nl/cgi-bin/primer3plus/primer3plus.cgi/) and verified for their specificity with Primer Blast (http://www.ncbi.nlm.nih.gov/tools/primer-blast/). In addition, two cucumber reference genes (18S and F-box) were selected from Shoresh et al. (2005) and Migocka and Papierniak (2011), respectively. All primer sequences appear in **Supplementary Table S1**. Each PCR amplification was performed for three independent biological repeats, with two technical repeats and carried out in a StepOnePlus™ RT‐qPCR (Applied Biosystems, USA), following the SYBR Green method (Fast SYBRTM Green Master Mix, Applied Biosystems) as described by Perrone et al. (2012). The PCR program consisted of an initial denaturation at 95°C for 10 min, followed by 40 cycles of 10 s at 95°C, 15 s at 60°C, and 20 s at 72°C. Gene expression data were calculated as expression ratios (quantity relative to that of control). The genes’ expression levels obtained by qRT-PCR, and those of the RNA-seq were correlated with linear regression.

To further quantify the CGMMV titer and *P. spinosum* concentration in the cucumber collar-region, cDNA from the samples of each treatment and time point were amplified by using CGMMV coat (Reingold et al., 2015) and movement protein gene primers, and *P. spinosum* specific primers for *Ubiquitin* and *Actin* (**Table S1**). All procedures were conducted as described above. For all primer sets, efficiency was determined by a standard curve.

### 2.8 *In situ* immunofluorescence labeling of CGMMV

Samples from the cucumber collar-region of CGMMV-infected plants (with or without *P. spinosum*), and from healthy control plants, collected on 13 dpvi, were sliced and fixed with 4% formaldehyde and 0.2% glutaraldehyde, as described previously (Reingold et al., 2015). The slices were washed twice with PBS-T (phosphate-buffered saline with 0.05% Tween-20), blocked using PBS with 1% milk (0% fat) for 30 min and incubated overnight at 4°C with IgG antibodies specific for CGMMV (Antignus et al., 1990; Antignus et al., 2001). Slices were washed twice with PBS-T, and the secondary antibody, goat anti-rabbit IgG-conjugated Alexa Fluor 488 (Invitrogen), was added to the slices in a 1:1,000 dilution in PBS followed by incubation at 37°C for 3 h. The slices were washed twice with PBS-T and kept in PBS in a sealed box. The fluorescence signal of the slices was detected using confocal microscopy (LSM510 Axiovert 100 M, ZEISS, USA).

### 2.9 Fluorescent *in situ* hybridization

FISH was conducted according to Gottlieb et al. (2006) with several modifications. Single-stranded DNA oligos of 20 nucleotides (5-GAACCAGTACGACCCTCCAA-3) were labeled with a Cy3 fluorophore and used as a probe. The probe (Hylabs, Israel) corresponded to the *P. spinosum Actin* gene. Collar-region samples were collected from *P. spinosum*-infected plants with or without CGMMV co-infection and from control plants, and were hand-sectioned, followed by fixation overnight at room temperature in Carnoy’s fixation (6:3:1 v/v/v chloroform: absolute ethanol: glacial acetic acid). Samples were decolorized twice in 6% H_2_O_2_ solution in absolute ethanol for 48 h, then washed twice in absolute ethanol and pre-hybridized with hybridization buffer (20 mM Tris–HCl, pH 8.0, 0.9% NaCl, 0.01% sodium dodecyl sulfate, 30% formamide) for 1 h at room temperature. Ten pmol fluorescent probe ml^-1^ was added to the hybridized samples followed by overnight incubation at room temperature. Samples were then examined using a confocal microscope (LSM510 Axiovert 100 M; ZEISS, USA).

## 3 Results

### 3.1 Core experiments for the study of late wilting disease using *P. spinosum* and CGMMV during single and co-infection

**Figure 1:**
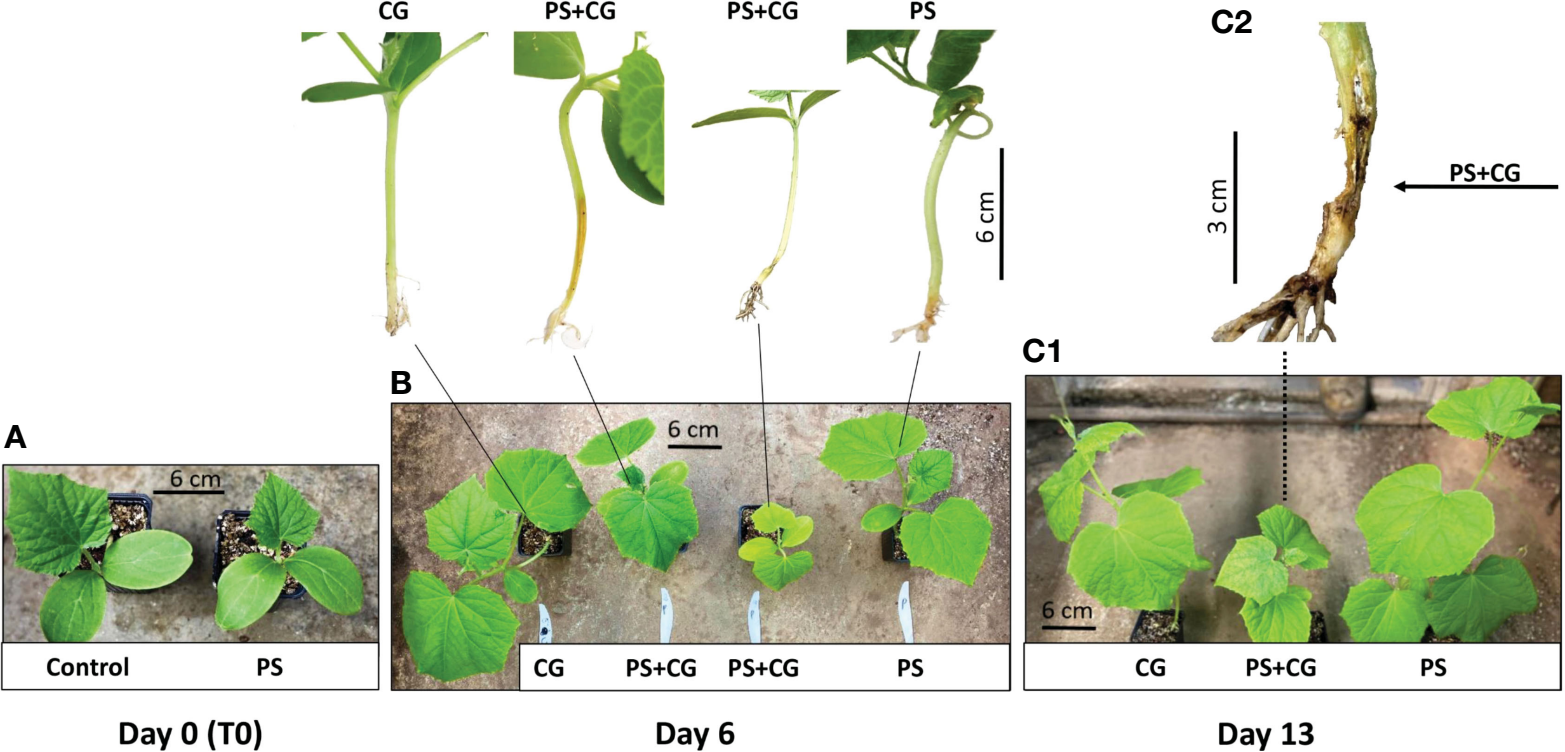
The influence of the co-infection process on cucumber plants at different time points after cucumber green mottle mosaic virus (CGMMV) inoculation. **(A)** Non-inoculated control and *Pythium spinosum* infected plant prior CGMMV inoculation (T0). **(B)** Two different growth constrained phenotypes of co-infected plants (P+CG) compared to CGMMV (CG) and *P. spinosum* (PS)-infected plants documented 6 days post CGMMV inoculation. (C1) Growth constrained phenotypes of co-infected plants (P+CG) compared with CGMMV (CG) and *P. spinosum* (PS) infected plants 13 days post CGGMV inoculation and (C2) collar region of a co-infected wilting plant (P+CG) 13 days post CGMMV inoculation.

Five days after the *P. spinosum* inoculation (which simulated the process of early damping-off stage with Pythium alone), 188 wilting cucumber plants were discarded; 100 inoculated plants and an additional 100 non-inoculated healthy control plants served the core experiments. Fifty cucumber plants from each group were then inoculated with CGMMV as described above, forming the four different treatments described above: (i) PS+CG; (ii) PS; (iii) CG, and (iv) C. The inoculation of the plants with CGMMV is considered as the start of the experiment (T0) and only minor growth constraints were observed in the *Pythium*-established plants (**Figure 1A**). No plant mortality was detected in any treatment until day 6 dpvi. The first wilting symptoms were observed only in the combined infection treatment (PS+CG) (**Figure 1B**), reaching 33.3% mortality between 6-13 dpvi (12 out of 36 plants) and the surviving co-infected plants suffered from severe growth inhibition (**Table 1**, **Figures 1B, C**). The first viral symptoms of mottle and mosaic appeared on day 10 and were well apparent on day 13 without any visual differences between the CG and PS+CG treatments. No wilting symptoms were observed in the PS, CG, and C treatments (**Table 1**, **Figures 1B, C**).

**Table 1:** The influence of the different infection treatments on cucumber plants, documented 13 days after cucumber green mottle mosaic virus (CGMMV) inoculation.

Treatment	C	CG	PS	PS+CG
**Mortality (%)**	0	0	0	33.3%*
**Plant height (cm)**	33.37 ± 2.02	28.56 ± 2.54	19.65 ± 0.69	11.43 ± 1.06*

Phenotypes include plants mortality incidence and plants height. (C) Non-inoculated control; (CG) CGMMV inoculated plants; (PS) *Pythium spinosum* infected plant; (PS+CG) co-infected plants with *P. spinosum* and CGMMV inoculation. Asterisk represents a significant difference between PS and PS+CG treatments at α=0.05. Student’s t-test was used for plant height comparisons and χ2 test for plant mortality incidence comparisons.

### 3.2 Cucumber RNA sequence data analysis

**Figure 2:**
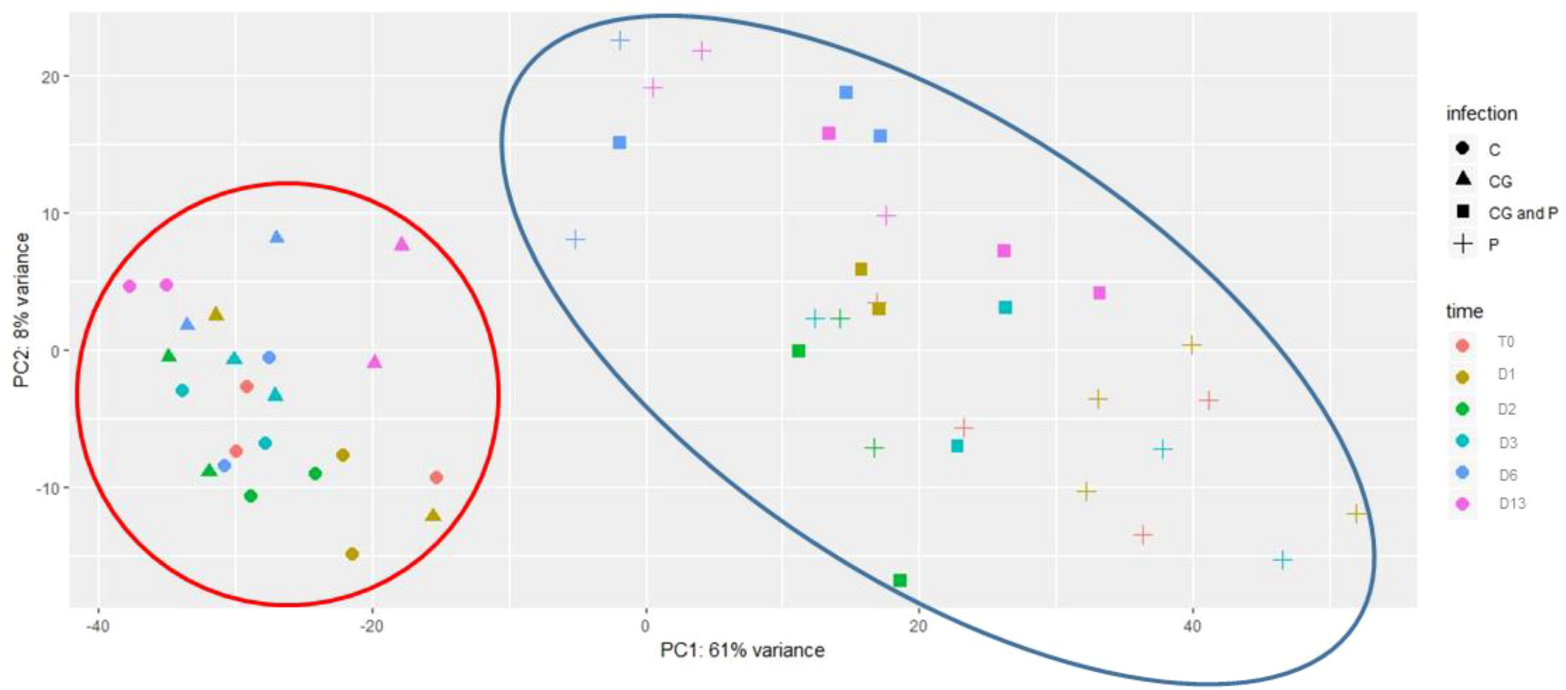
Principal component analysis (PCA) of gene expression levels in cucumber plants including all treatments. : un-inoculated plants (c); inoculated with Cucumber green mottle mosaic virus (CGMMV) (CG); inoculated with CGMMV and *Pythium spinosum* (CG and P); and, inoculated solely with *P. spinosum* (P). Samples were taken at time points starting with pre-infected samples with *P. spinosum* (TO), and at various days (D) after the infection with CGMMV (D1, D2, D3, D6, D13). The first two principal components are plotted. Shapes indicate different treatments. Colors indicate different times. The clusters containing control and CGMMV-infected plants appear in the large red circle, treatments including plants infected with Pythium and CGMMV+Pythium appear in the large blue circle. Percentages of variation explained by each PC are indicated along the axes.

To gain insight into the genes involved during the infection process of *P. spinosum* followed by CGMMV infection in cucumber plants, samples of collar-region from the four treatments (P, P+CG, CG, C) were collected in six-time points: pre (T0) and post-CGMMV inoculation (1, 2, 3, 6, and 13 days). Data obtained from the transcriptomic analysis presented an average of 26,936,546 reads per sample (**Table S2**). High quality reads (97%) were mapped to *C. sativus* CoDing Sequences (CDS) reference. Overall, 20,792 different transcripts were identified (**Table S3**). A principal component analysis (PCA) of all collar-region transcriptional changes divided the samples into two main clusters. The first cluster includes 23 samples from all the healthy control plants and also from the plants infected only with CGMMV; the second cluster includes all 30 samples infected with *P. spinosum* with and without CGMMV, regardless of the sampling time. Most of the variance was defined by PC1 (61%) and PC2 (8%) (**Figure 2**).

### 3.3 Gene expression profiles during inoculation with each pathogen separately

#### 3.3.1 DEGs in surviving plants infected with *P. spinosum* demonstrated increased defense mechanisms against a necrotrophic pathogen

To study the molecular mechanisms of plants infected with *P. spinosum* prior to CGMMV inoculation (T0), transcriptomic data from plants 5 dpi with *P. spinosum* alone were compared to control plants. Analysis of the transcriptional profile (Padj < 0.01, -2 > log2fold > 2) revealed 1,034 DEGs, of which 657 genes were upregulated, and 377 genes were downregulated (**Table S4**).

**Figure 3:**
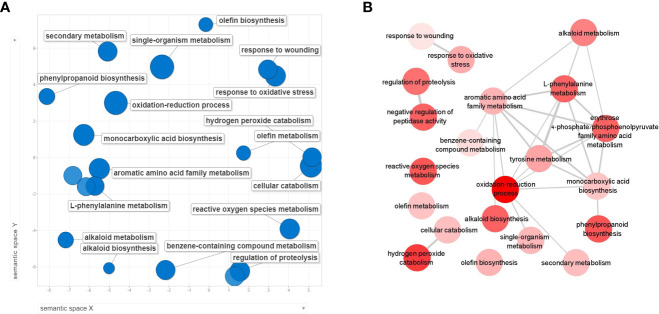
REVIGO scatterplot visualization of Gene Ontology (GO) analysis based on differentially expressed genes (DEGs) from collar region of cucumber plants 5 dpi with *Pythium spinosum* vs healthy control plants. Functional classification of the 657 upregulated genes **(A)** were assigned into biological process GO term that were significantly enriched (False Discovery Rate <0.05). Circle size represents the -log10 transformed FDR in REVIGO analysis. **(B)** Interactions between the upregulated biological process categories. Darker circle color represent lower –log10 of the P value.

**Figure 4:**
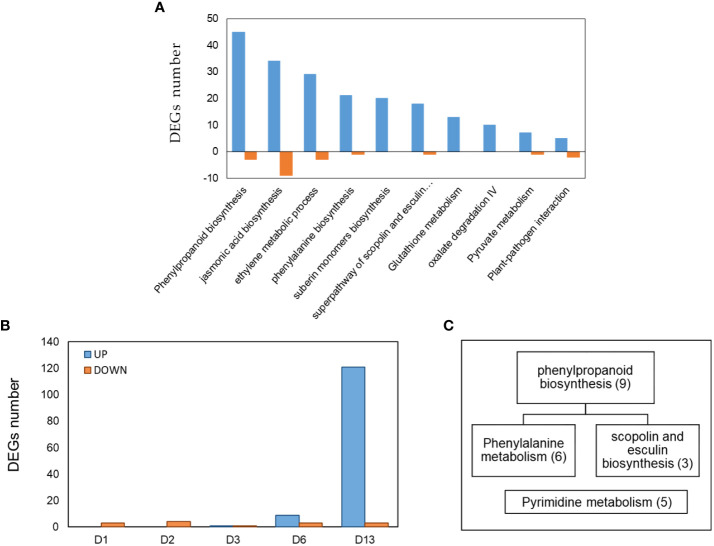
**(A)** Pathway enrichment analysis (FDR < 0.05) of DEGs obtained from cucumber plants infected with *Pythium spinosum* vs healthy control plants. Data was obtained from KEGG and Cucurbit Genomics Database. **(B)** The distribution of 143 differential expression genes (DEGs) obtained at different days (D1, D2, D3, D6, D13) from cucumber plants infected with Cucumber green mottle mosaic virus (CGMMV) vs healthy control plants. **(C)** Assignment of the DEGs obtained from day 13 (i.e., **B**) into enriched pathways (*P* < 0.05) based on KEGG annotation. Number of genes in each enriched pathway appears in parenthesis.

When assigning the 1,034 DEGs into Clusters of Orthologous Groups of proteins functional classification, 80.8% were annotated into 20 categories. The largest identified groups were secondary metabolite biosynthesis (11.3%) followed by carbohydrate transport and metabolism (11%), transcription (10.7%), signal transduction mechanisms (9.3%) and defense mechanisms (1.5%). All these groups also relate to plant immunity (McDowell and Dangl, 2000; Trouvelot et al., 2014; Zaynab et al., 2018), (**Figure S2**). To further identify the biological processes involved in the cucumber collar-region infected with *P. spinosum*, the DEGs were assigned into GO enrichment analysis (**Table S5**). The most significant upregulated GO terms were demonstrated to be plant defense patterns under pathogenic attack. These GO terms included a single organism metabolism, oxidation-reduction processes, cellular catabolism, L-phenylalanine metabolism and phenylpropanoid biosynthesis also related to defense mechanisms against necrotrophic pathogens (Munir et al., 2019) (**Figure 3A**). From the downregulated genes, only oxidation-reduction processes and pathogenesis were enriched. By examining the upregulated GO groups, interactions between the major categories were observed (**Figure 3B** **)**. Pathway enrichment based on KEGG and CuGenDB showed nine main upregulated pathways highly related to defense mechanisms, which include the phenylpropanoids (number of DEGs (n)=45), ethylene metabolic process (n=29), jasmonic acid (n=34) and superpathways of esculin and scopolin biosynthesis (n=19) (**Figure 4A**).

Moreover, several gene families related to defense against necrotrophic pathogens were differentially expressed. These groups included PR proteins: [PR-2 (n=1), PR-3 (n=6), PR-5 (n=3), PR-9 (n=23), PR=15 (n=9)]; expansin (n=9) and transcription factor families associated with defense responses, including: LOB (n=8) MYB (n=8), NAC (n=12), WRKY (n=9) (**Table S4**). Members of those families regulate gene expression in response to biotic stimulation including oomycetes (Thatcher et al., 2012; Yang et al., 2019). In addition, several DEGs related to plant hormone signaling involved with plant defense and growth were downregulated, included auxin (5 up and 10 down excluding LOB), gibberellin (n=4), and brassinosteroids (n=4) (**Table S4**).

#### 3.3.2 DEGs at the collar region in response to CGMMV leaf inoculation were initially apparent at 13 dpvi

The comparison of collar region samples of CGMMV-infected plant vs non-infected plants (Padj < 0.01, -2 ≥ log2fold ≥ 2) reveals that a small number of DEGs were represented during the first six days post-inoculation (11 downregulated and ten upregulated) and none of them was GO-enriched. However, on day 13, a significantly larger number of DEGs were upregulated compared to earlier (121 upregulated and three downregulated from a total of 143 DEGs among all time points) (**Table S6**). Enriched pathways were obtained only on day 13, and DEGs were assigned to phenylpropanoid biosynthesis/phenylalanine and pyrimidine metabolism, which are related to SA signaling and defense against biotrophic pathogens (**Figures 4B, C**) (Zhang et al., 2014; Liu et al., 2020). Additional DEGs related to plant-pathogen interaction such as kinase protein receptors, calmodulin, WRKY family genes, RNA dependent RNA polymerase, brassinosteroids and UPD-glycosyltransferase (Du et al., 2009; De Bruyne et al, 2014; Leibman et al., 2018) were observed.

### 3.4 Rapid reduction in DEGs related to defense mechanisms against necrotrophic pathogens is exhibited in Pythium and CGMMV co-infection

**Figure 5:**
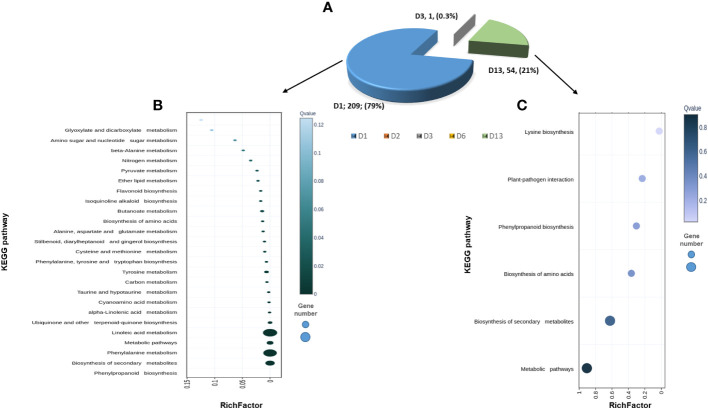
**(A)** Pie diagram of 263 differentially expressed gene (DEGs) obtained from cucumber plants infected with *Pythium spinosum* and Cucumber green mottle mosaic virus (CGMMV) vs *P. spinosum* alone. **(B)** Scatterplot of 209 DEGs that have changed one day (D1) post-CGMMV-inoculation assigned into the top 25 KEGG pathways. **(C)** Scatterplot of 54 DEGs that have changed 13 days after CGMMV infection divided into the top 5 KEGG pathways. Rich Factor is the ratio of differentially expressed gene numbers annotated in this pathway terms to all gene numbers annotated in this pathway term. Coloring indicates q-value, a lower q-value indicates more significant enrichment, and the point size indicates the DEGs number. • *q ≤* 0.05 as significantly enriched. • * Annotated from KOBAS, Kobas.cbi.pku.edu.cn.

To reveal the changes that occurred in *P. spinosum*-infected plants following CGMMV inoculation, plants co-infected with CG+PS vs PS alone for each time point were compared (1, 2, 3, 6 and 13 dpvi). In total, DEGs were downregulated that related to defense mechanisms against necrotrophic pathogens were detected 1 dpvi (of *P. spinosum*-infected plants). 263 DEGs with (P_adj_ ¾ 0.01, -2 ≥ log_2_fold ≥ 2) were detected, among them, 209 DEGs already differentially expressed as early as 1 dpvi, of those 206 DEGs were downregulated. No major changes in DEGs were detected during days 2-6. However, on day 13, 52 DEGs were upregulated while only two DEGs were downregulated (**Figure 5A**, **Table S7**).

While assigning the DEGs to the KEGG pathway analysis, among the top 25 pathways, 21 were enriched (*P* < 0.05) and were down-regulated on 1 dpvi) **Figure 5B**). The most significant number of DEGs were assigned to ‘phenylpropanoid biosynthesis’ (n=24), ‘biosynthesis of secondary metabolites’ (n=46), ‘phenylalanine metabolism’ (n=13) and ‘linoleic acid metabolism’ (n=4) KEGG pathways (**Figure 5B**), all belonging to defense mechanisms against pathogens. In contrast, the KEGG analysis of the upregulated DEGs from day 13 were only enriched for ‘lysine biosynthase’ pathway (n=1) (**Figure 5C**). However, additional DEGs were assigned to the non-enriched pathways of ‘plant-pathogen interactions’ (n=1), ‘phenylpropanoid biosynthesis’ (n=1) and ‘biosynthesis of secondary metabolites’ (n=2) (**Figure 5C**).

**Figure 6:**
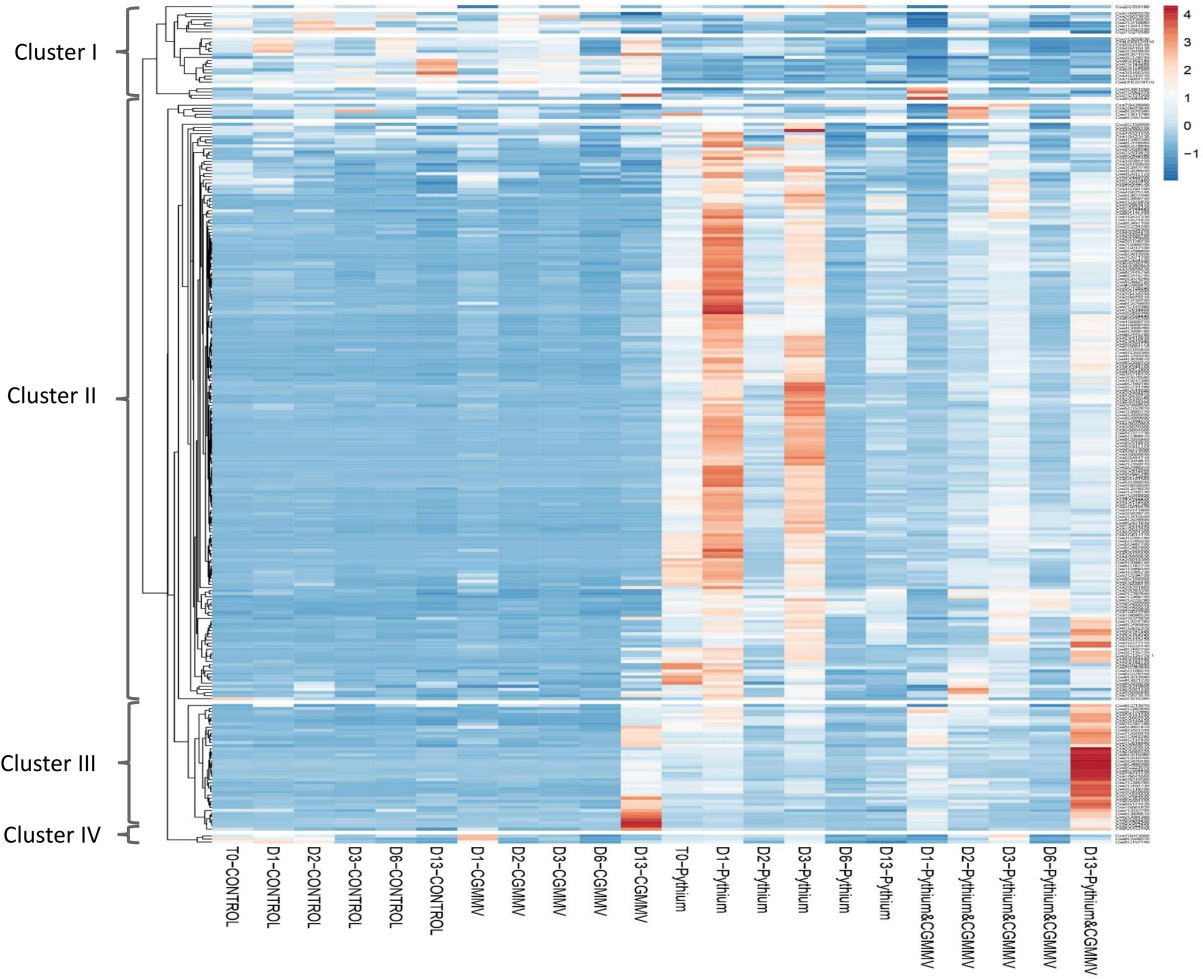
Heatmap that represents the expression profiles of 263 differential expressed genes (DEGs) obtained from the comparison of cucumber plants co-infected with Cucumber green mottle mosaic virus (CGMMV) and *Pythium spinosum* vs plant infected with *P. spinosum*. Each column represents different treatment (non-infected plants-CONTROL; CGMMV; *P. spinosum*; and *P. spinosum* and CGMMV) at different time points, starting from T0 (*Pythium*; control) and at different points post infection with CGMMV (D1, D2, D3, D6, D13). Each column represents the average of the replications (up to four) from each treatment. The list of the DEGs in each cluster is also described in **Table S8**.

We compared the 263 specific DEGs derived from CGMMV inoculation of established-*Pythium* infected plants (PS+CG) for all of the 53 experimental samples. Three main patterns were detected by using the expander software (**Figure 6**, **Table S7**). Cluster 1 included 26 genes that were downregulated within all samples with *Pythium* (with/without CGMMV) vs. controls and CGMMV alone. There was no distinctive pattern for these genes.

Cluster 2, including 181 genes mostly not expressed in the control or CG plants. However, the cluster had a clear high expression pattern in plants infected with *Pythium* alone. Interestingly, 118 DEGs were strongly downregulated 1 dpvi (**Figure 6**, **Table S8**). The cluster 2 pattern was constant for most DEGs throughout the 13 days in plants infected with both CGMMV and *Pythium*.

**Figure 7:**
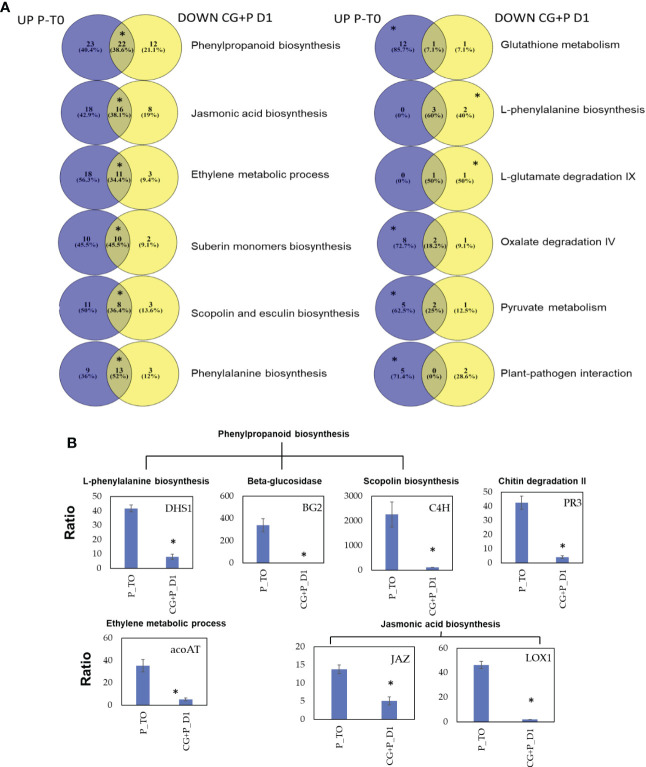
**(A)** Venn diagrams describing the pathway enrichment of DEGs obtained from cucumber plants before and after the CGMMV inoculation of plants infected with *P. spinosum*. Purple colors representing DEGs that upregulated in plants infected with *P. spinosum* alone; yellow color represent DEGs that were downregulated 24* h* after CGMMV inoculation of *P. spinosum*-infected plants. In each pathway, the position of the asterisk represents whether the pathway was enriched (for the upregulated (left), downregulated (right) or both (middle)). **(B)** Relative qPCR validation of selected genes from main enriched pathways that were upregulated in cucumber plants infected with *P. spinosum* alone and later downregulated 1 dpi with CGMMV. Asterisks denote significant differences (*P* < 0.05).

Eight enriched pathways were obtained from the 181 DEGs of cluster 2. To learn the direct influence of the CGGMV penetration into the established-*Pythium* infected plants, the eight enriched pathways were compared to the 10 enriched pathways derived from plant infected with *Pythium* alone (prior to CGMMV inoculation, i.e. **Figure 4A**). Six shared enriched pathways were obtained, which included mutual DEGs that were upregulated with *Pythium* alone, and strongly down regulated after the CGMMV inoculation (**Figure 7A**). All of these six mutual pathways are involved in plant defense mechanisms against necrotrophic pathogens including phenylpropanoid (22 mutual DEGs); jasmonic acid (16); phenylalanine (13); ethylene (11); suberin monomer biosynthase (10); scopolin and esculin biosynthesis (8). In addition, two pathways, L- phenylalanine (5 DEGs) and L-glutamate (2 DEGs) were enriched only during combined infection (**Figure 7A**). Among the 10 enriched pathways, four were solely upregulated with *Pythium* alone [glutathione metabolism (13 DEGs), oxalate degradation (10), pyruvate metabolism (7) and plant pathogen interaction (5)]. Although these four last pathways were not enriched during the co-infection, they also include DEGs that were downregulated at 1 dpvi (**Figure 7A**).

Relative expression of 7 selected down-regulated genes known to be related to defense pathways against necrotrophic pathogens [JA, phenylpropanoid, scopolin, ethylene and plant-pathogen interaction] were validated. The qRT-PCR results confirmed the significant down-regulation (*P* < 0.05) for all the seven selected genes 1 dpvi vs established*-Pythium* infected plants (prior to CGMMV inoculation, **Figure 7B**). Furthermore the qRT-PCR results also correlated well with the RNA-Seq data analysis (R^2 = ^0.807, *P* < 0.05, **Figure S3**).

Cluster 3, includes 42 genes upregulated only 13 dpvi. Moreover, all the 42 genes were differentially expressed in the combined infection, while 26 of them were also differentially expressed in CG plants (**Table S8**). These genes are mostly related to defense mechanisms against viral infection including RdRp (2 DEGs), WRKY transcription factors (5 DEGs) and pyrimidine metabolism (5 DEGs). RNA-dependent RNA polymerase (RdR1c1) and two selected genes from pyrimidine metabolism:AAA-ATPase (BCS1-ATOM66) and ATP-dependent zinc metalloprotease ftsH (BCS1) were validated by qRT-PCR (**Figure S4**). All these genes are upregulated following CGMMV infection (13 dpvi), and therefore this data accords with the RNA-Seq data analysis (R^2 = ^0.986, *P* < 0.05). An additional minor fourth Cluster included 3 genes with an unspecified pattern (**Table S8**).

**Figure 8:**
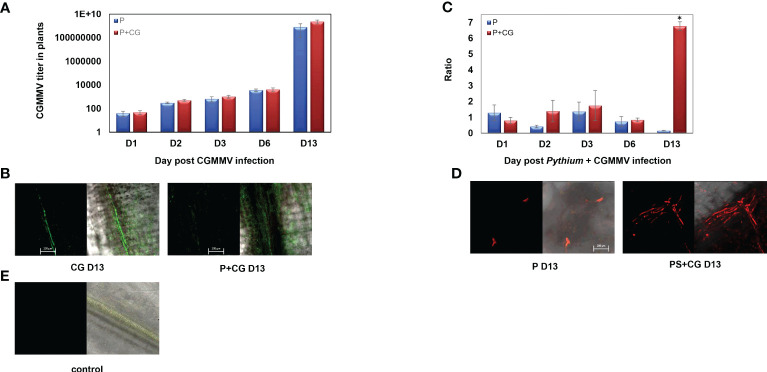
**(A)** Relative gene expression ratio of Cucumber green mottle mosaic virus (CGMMV) titer in collar-region of cucumber plants inoculated with CGMMV or with combined infection of CGMMV and *Pythium spinosum* at 1, 2, 3, 6, 13 days after the CGMMV inoculation. The S.E.M. is indicated on each bar. **(B)** Representative confocal microscopy images of *in situ* immunofluorescence labeling (FITC, 10 pmol) of CGMMV in cucumber collar-regions with or without *P. spinosum* on day 13. **(C)** Relative gene expression ratio of *P. spinosum Actin* gene in cucumber collar region inoculated with *P. spinosum* alone or with combined infection of CGMMV and *P. spinosum* at days 1, 2, 3, 6, 13 after the CGMMV inoculation. The S.E.M. is indicated on each bar. Asterisks represent a significant difference (α = 0.05). **(D)** Representative confocal microscopy images of fluorescent *in situ* hybridization labeling (Cy-3, 10 pmol) of *P. spinosum Actin* gene in cucumber collar-regions with or without CGMMV inoculation in day 13. **(E)** Healthy control plant. [Bar = 200 μm]. For the confocal microscopy images, we provided the two captured images of the bright field with the florescent channels red or green respectively (right side) and the florescent signal alone (Left side).

CGMMV titer in the collar-region increased from day 6 to day 13, with or without the presence of *P. spinosum*, indicating that *P. spinosum* did not affect CGMMV titer (accumulation or decrease) in co-infected plants (**Figure 8A**). This was validated by the *in situ* immunofluorescence analysis on 13 dpvi, as no substantial differences were visualized between CGMMV vs PS+CG plants (**Figure 8B**). In contrary, relative gene expression ratio of the *P. spinosum*-*Actin* in the collar-region shows that in plants infected with *P. spinosum* alone, the *Actin* concentration remained constant during the next 13 days compared to the *Actin* concentration at T0. However, a 36-fold increase of *P. spinosum*-*Actin* was detected at 13 dpvi in plants co-infected with *P. spinosum* and CGMMV when compared to plants inoculated with *P. spinosum* alone (**Figure 8C**); similar results were also detected for the *P. spinosum-Ubiquitin* (**Figure S5**). FISH analysis of the *P. spinosum-Actin* gene also visually demonstrated the increase of the *P. spinosum* hyphae in the PS+CG plants when comparing to PS infected plants (collar region) on 13 dpvi (**Figure 8D**).

The results support our hypothesis and confirm the scenario of a unique situation *in planta* that provided optimal conditions for re-proliferation of *P. spinosum* during co-infection.

## 4 Discussion

The current work demonstrated the complexity of plant disease that involves infection with more than one pathogen. The disease complex yield different epidemiological and phenotypic outcome that correlated with different transcriptomic dynamics for each infection scenario.

While inspecting the transcriptomic results 5 dpvi with *P. spinosum* (i.e., at T0), we relate to plants just before the penetration of the CGMMV into the system. At this stage, those plants already had survived the damping-off stages and should further develop with *P. spinosum* as a minor pathogen in their tissues, i.e., minor growth reduction but without extensive damage or wilting (Woltz, 1978). At this stage, these plants exhibit a strong reaction against necrotrophic pathogens, emphasize by the increase of secondary metabolites that includes phytohormones, phytoalexins and physical defense pathways (Stringlis et al., 2019). While the results include a significant upregulation in DEGs related to the classic phytohormone responses of JA and ET (Wasternack and Song, 2017), phenylalanine ammonia-lyase is also upregulated in concordance with previous findings of *Pythium* infections (Oliver et al., 2009; Verbeek et al., 2019). In addition, DEGs in the pathway of the phytoalexin scopolin, a secondary metabolite, are also upregulated. This phytoalexin belongs to the coumarin group, which includes important natural compounds that supply the plant with antimicrobial and antioxidative activities (Siwinska et al., 2014). This synthesis starts with UDP-glucosyltransferases that lead to the phenylpropanoid pathway, a key element in defense mechanisms against various biotic stresses (Sun et al., 2014; Stringlis et al., 2019). In the phenylpropanoid pathway, PAL leads to synthesis of scopolin along with cinnamate 4-hydroxylase (CH4) and β-glucosidase (Chong et al., 2002).

PRs proteins play a substantial role in plant defense against necrotrophic pathogens (Van Loon, 1997). Among the upregulated genes encoding the PRs proteins, the gene encoding the peroxidase (PR-9) was highly significant, and besides its role in reactive oxygen species (Matić et al., 2016) PR-9 is also involved in the formation of physical barriers against pathogens, including suberin formation in response to stresses such as wounding and pathogens (Kashyap et al., 2021). In addition to the above-enriched pathway, genes encoding the transcription factors were upregulated, e.g., WRKY, Ethylene Responsive Factor (ERF), NAC (NAM), MYB, Basic leucine zipper domain (bZIP), and Basic helix-loop-helix (bHLH) families. These families play an important role in response to a range of biotic stresses including oomycetes, fungi, and bacteria and are a key downstream transcriptional regulator of JA and ET signaling (Thatcher et al., 2012; Li et al., 2019).

The established-*Pythium* infected plants (that survived early damping-off) mostly suffered from growth restrictions. While *Pythium*’s direct damage to root systems hampered plant growth (**Figure 1A**), the downregulation of DEGs in several hormonal pathways related to plant growth was also apparent. Under multiple biotic and abiotic stresses, plant hormones assign limited resources to respond to the most severe stress and develop several signaling pathways to regulate the balance between different defense responses and plant growth (Matyssek et al., 2005; Yang et al., 2019). During the infection with necrotrophic pathogens, plants shift their resources into the defense response by increase the JA/ET pathway; meanwhile the activities of the BR, auxin and GA signaling pathways related to plant growth are reduced (Boller and He, 2009; Dou and Zhou, 2012). Several studies showed that JA does not work independently, but rather acts in a complex signaling network with other plant hormone signaling pathways, especially in the cross-talk of JA–auxin, JA-BR, and JA-GA signaling pathways (Pérez and Goossens, 2013; Goossens et al., 2016; Yang et al., 2019), which probably contributed to the limitation of plant growth.

Before providing insight into the consequences of the CGMMV inoculation of the *Pythium* pre-infected system, an inspection of the plant response to individual infections is needed. From the PCA results (**Figure 2**), it is apparent that healthy plants and plants infected with CGMMV alone are clustered together, suggesting a minor influence of the CGMMV infection on the gene expression pattern of the collar-region. Temporal inspection of the plant response to the CGMMV infection in the collar-region shows that the plant reaction to the virus starts elevating from 6 dpvi; however, only at 13 dpvi was a significant response revealed. At 13 dpvi, the CGMMV titer also becomes highly significant in the collar-region (**Figures 8A, B**) simultaneously with typical leaf mottle and mosaic symptoms (Philosoph et al., 2019). Our findings and additional studies suggest that the local molecular response to the CGMMV infection only occurs when the viral titer becomes significant, demonstrating the local influence of the viral infection in the collar region. These results in increased DEGs that relate to pathways involved in defense mechanism such as phenylpropanoid (Li et al., 2017; Sun et al., 2019), including specific upregulated genes relating to biotrophic defense and plant response to a viral infection such as tobacco glucosyltransferase (TOGT) (Chong et al., 2002) and RNA dependent RNA polymerase associated with the antiviral RNA silencing pathway (Leibman et al., 2018; Molad et al., 2021). The upregulation of gene BCS1-ATOM66 (AAA-ATPase 1-like protein) can be related to cell death and SA signaling (Zhang et al., 2014) but interestingly also to pyrimidine metabolism, known to be increased during viral infection in humans (DeVito et al., 2014).

### 4.1 Rapid decrease in plant immunity response against a necrotrophic pathogen following inoculation by the biotrophic pathogen

In contrast to the late reaction in the plant collar-region in response to CGMMV inoculation into naive plants, CGMMV inoculation into established-*Pythium* plants elicited a rapid host response as early as 1 dpvi. Moreover, insight into the specific DEGs of this co-infection scenario revealed a sharp downregulation in several pathways related to plant immune responses to the necrotrophic pathogen. Moreover, while comparing those DEGs to those upregulated in the *Pythium*-established plants (on T0), not only those DEGs involve the same pathways, but also included a large number of mutual genes (**Figure 7**). Some of these pathways such as JA/ET are known to take part in cross-talk with SA (Koornneef and Pieterse, 2008).

CGMMV is a biotrophic obligate parasite, that stimulates plant responses through the SA pathway (Yang et al., 2015), while *Pythium* is a necrotrophic pathogen. Previous studies show that simultaneous activation of both SA and JA/ET pathways in the same host is mostly exhibited by suppressing the JA/ET pathway (Spoel et al., 2007; Pieterse et al., 2012). Although we do not observe any rapid response in the plant collar-region to the biotrophic pathogen inoculated in the leaf (e.g., SA pathway-related genes), a rapid suppression of pathways involved in the response against necrotrophic pathogens was apparent. This may suggest that the cross communications occur and result from systemic reactions, as previously described by Caarls et al. (2015). Their review described potential regulatory mechanisms that suppress the JA pathway in the presence of SA *via* several transcription factor genes, such as specific members of the bHLH and WRKY family (Caarls et al., 2015; Aerts et al., 2021).

### 4.2 Major differences in temporal changes between single inoculation and co-infection

While following the pattern of the 263 DEGs obtained from the co-infected plants, throughout all the treatments in the experiment period, three main dynamic patterns are observed that were corroborated with each phenotypic characterization of the plant responses. Genes in *Pythium*-established plants kept relatively high expression level until 6 dpvi (11 days after *Pythium* inoculation), but with fluctuation between time points (**Figure 6**), a phenomenon previously observed in another *Pythium*-infected pathosystem (Shin et al., 2014; Shin et al., 2016). From 6 dpvi onward, those DEGs showed a milder expression pattern. This molecular pattern is in line with the phenotypic aspects, as these *Pythium*-established plants only showed moderate growth constraints and very limited mortality incidence in our experiments and in previous studies (Philosoph et al., 2018).

The co-infected plants demonstrated two other patterns. The second pattern involved a group of DEGs related to the defense mechanisms against necrotrophs and demonstrated a sharp downregulation as early as 24 hours post-CGMMV inoculation. Most of these genes maintained a relatively low expression throughout the experiment, although several genes were suddenly upregulated 13 dpvi. The phenotype of the co-infected plants supported the gene response pattern, demonstrating severe stress symptoms, including 30% mortality, while the rest of the plants suffered from extreme growth constraints several days before the appearance of the viral symptoms (**Figure 1B**). The third pattern involved genes that were upregulated only 13 dpvi. Most of these genes were expressed with the same pattern as in plants infected with CGMMV alone. This pattern was well correlated with a significant amount of viral titer quantified in the collar-region (**Figure 8A**), along with mottle and mosaic symptoms that appeared 13 dpvi (**Figure 1C**). These data are supported by *in situ* hybridization (**Figure 8B**), all the results supporting the claim that *Pythium* does not influence directly CGMMV accumulation.

Quantification of *Pythium* in the plants reveals that the pathogen’s concentration significantly increased on 13 dpvi vs PS (**Figure 8C**) and supported by the FISH results (**Figures 8D, E**). These results may explain the plant collapse after the co-infection process. Moreover, several upregulated DEGs 13 dpvi by the co-infected plants are related to defense mechanisms against necrotrophic pathogens such as ACC, LOX and phospholipase a1 known to be stimulated by the JA/ET pathway (Van Loon et al., 2006; Wasternack and Song, 2017). These signs of recovery in the plant defense may imply a renewed battle of the host against *Pythium*, after the destabilization following CGMMV infection. It also opens a new research question about the boundaries of the time windows examined and the ability to depict plant susceptibility. The importance of such a time window can significantly contribute to optimizing pest management against co-infection. For example optimizing anti-pythium compounds application timing to just before the agricultural practices that spread the CGMMV (triseling or leaf cutting), or application of compounds that increases plant defense against necrotrophic pathogens (Dempsey et al., 2022; Yu et al., 2022), during the most sensitive stages.

## Data availability statement

The datasets presented in this study can be found in online repositories. The names of the repository/repositories and accession number(s) can be found in the article/**Supplementary Material**.

## Author contributions

AP: conducted the plant and molecular experiments and bioinformatics analysis and lead the writing of the manuscript. NL: conducted the FISH experiment. NS: genome assembling and bioinformatics. YE: experimental design. AD and OF: experimental design, guidance, participate in manuscript writing. All authors contributed to the article and approved the submitted version.
