# Supplementary figures and images for: Rapid defense mechanism suppression during viral- oomycete disease complex formation
Source: Front Plant Sci. 2023 Jun 9;14:1124911. doi: 10.3389/fpls.2023.1124911 (PMC10288809; doi:10.3389/fpls.2023.1124911)

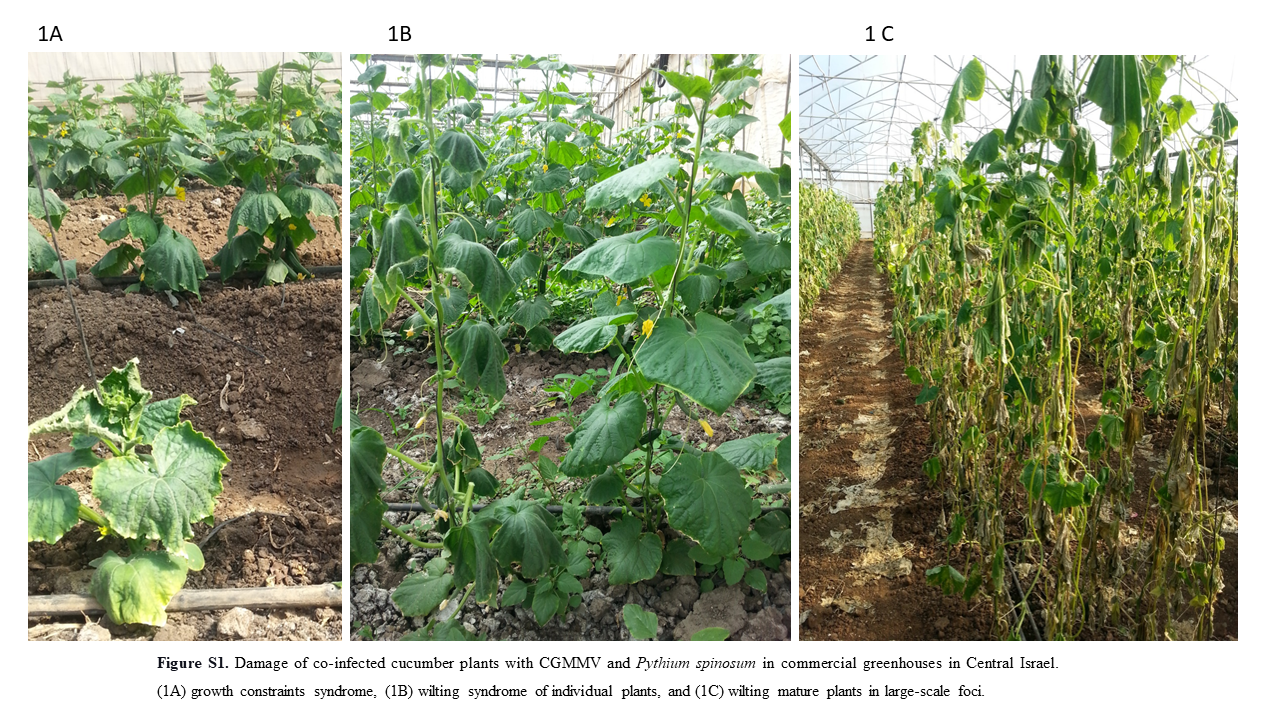

Supplement: Supplementary file 1 [file Image_1.tif]

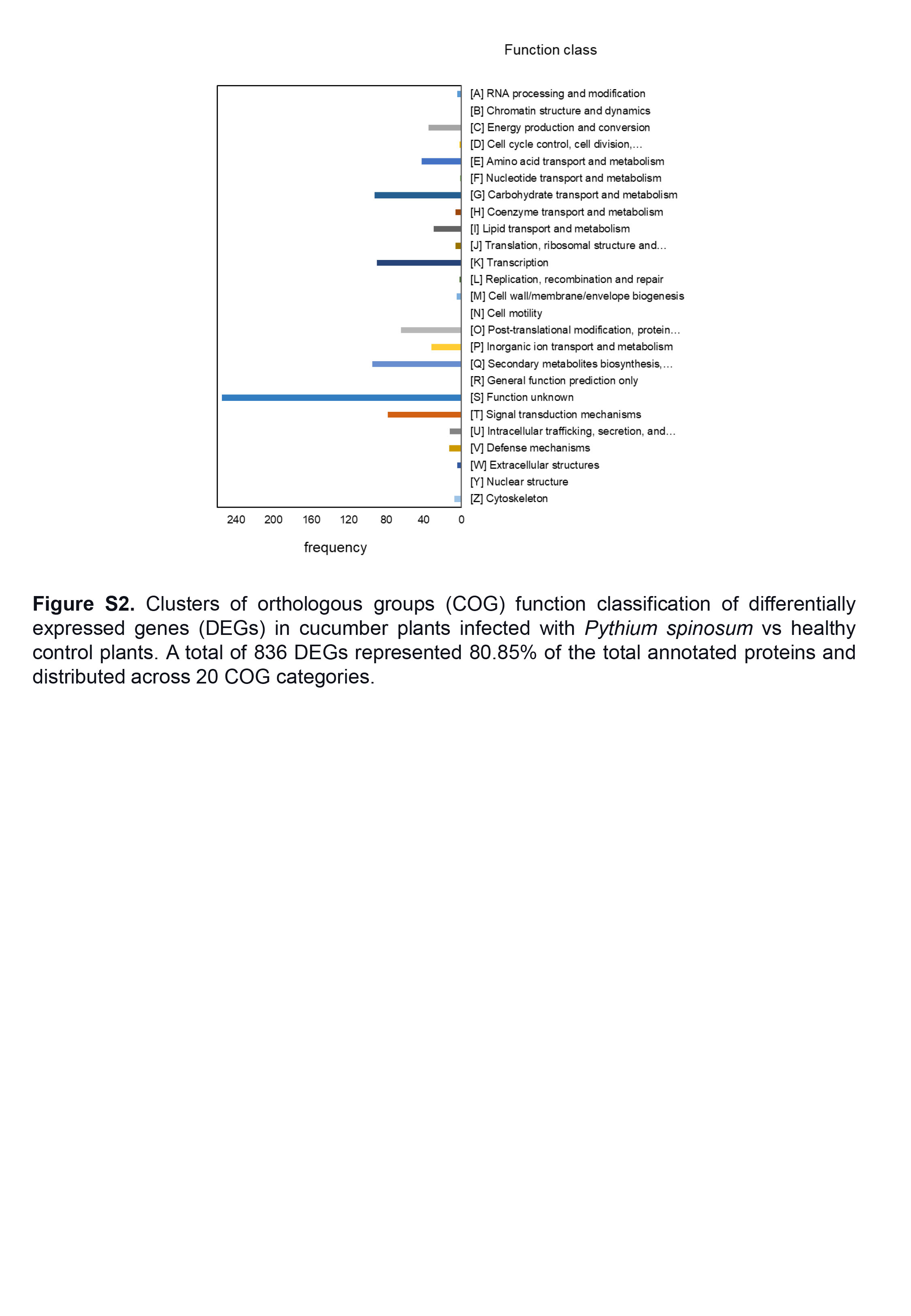

Supplement: Supplementary file 2 [file Image_2.jpeg]

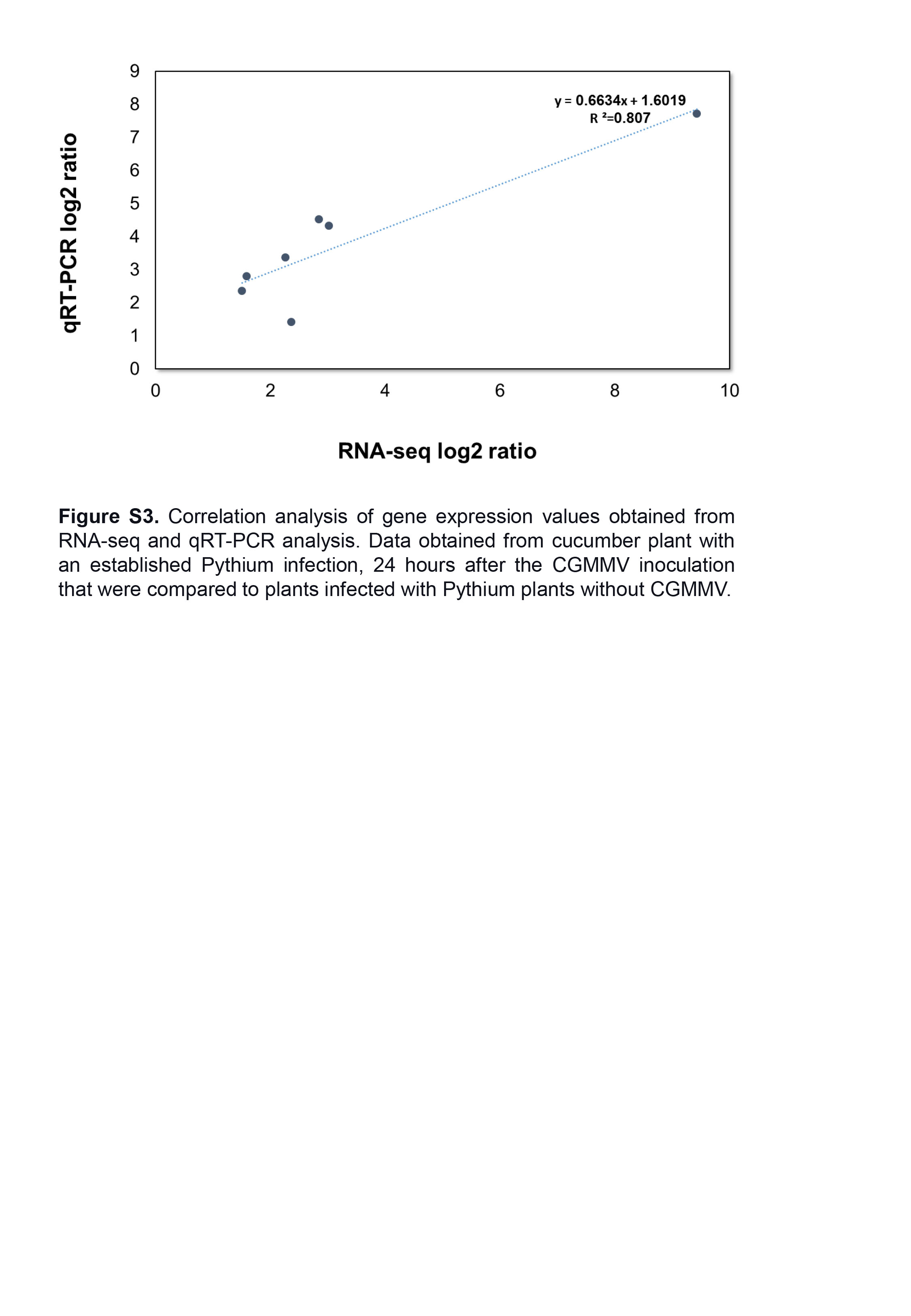

Supplement: Supplementary file 3 [file Image_3.jpeg]

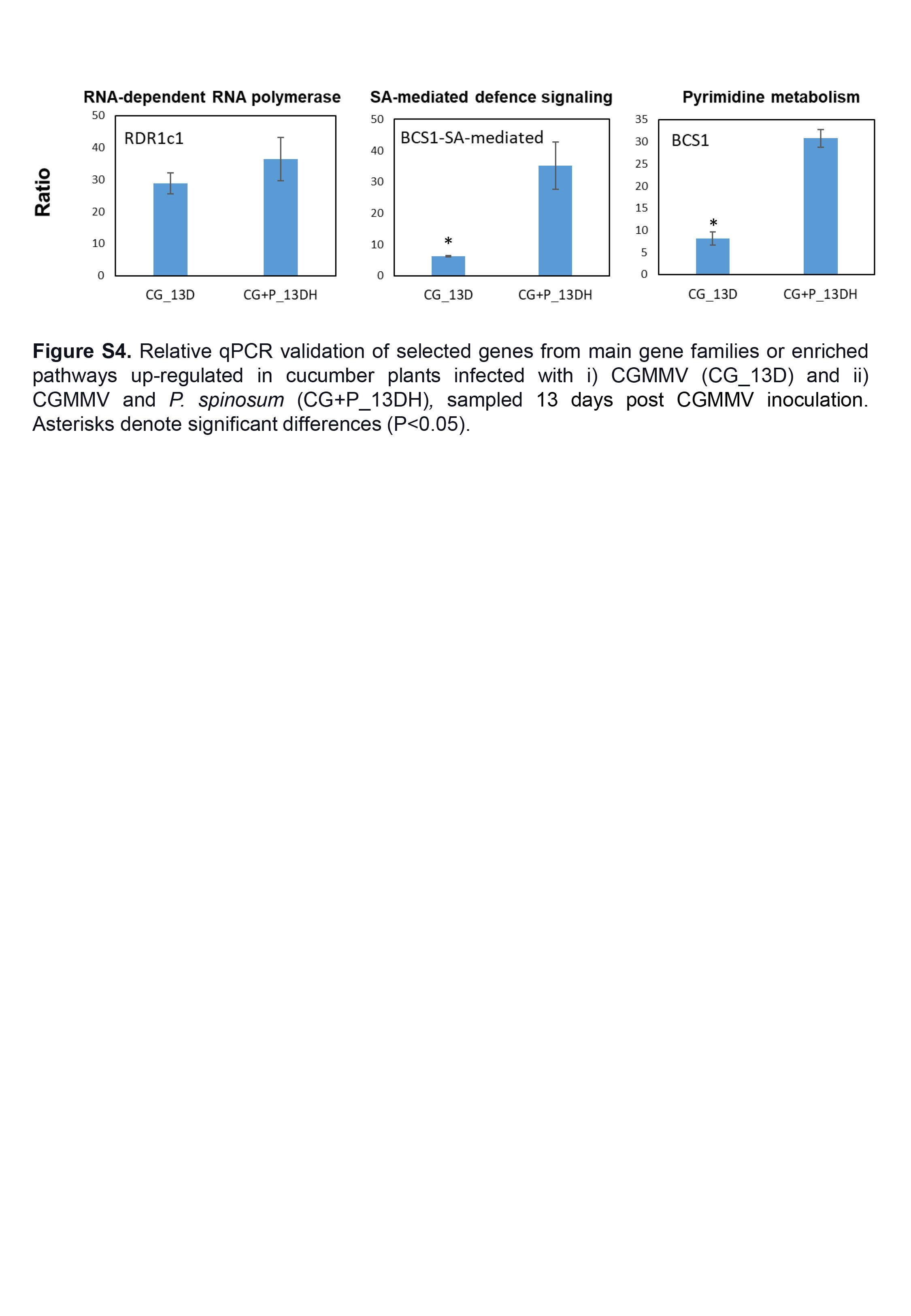

Supplement: Supplementary file 4 [file Image_4.jpeg]

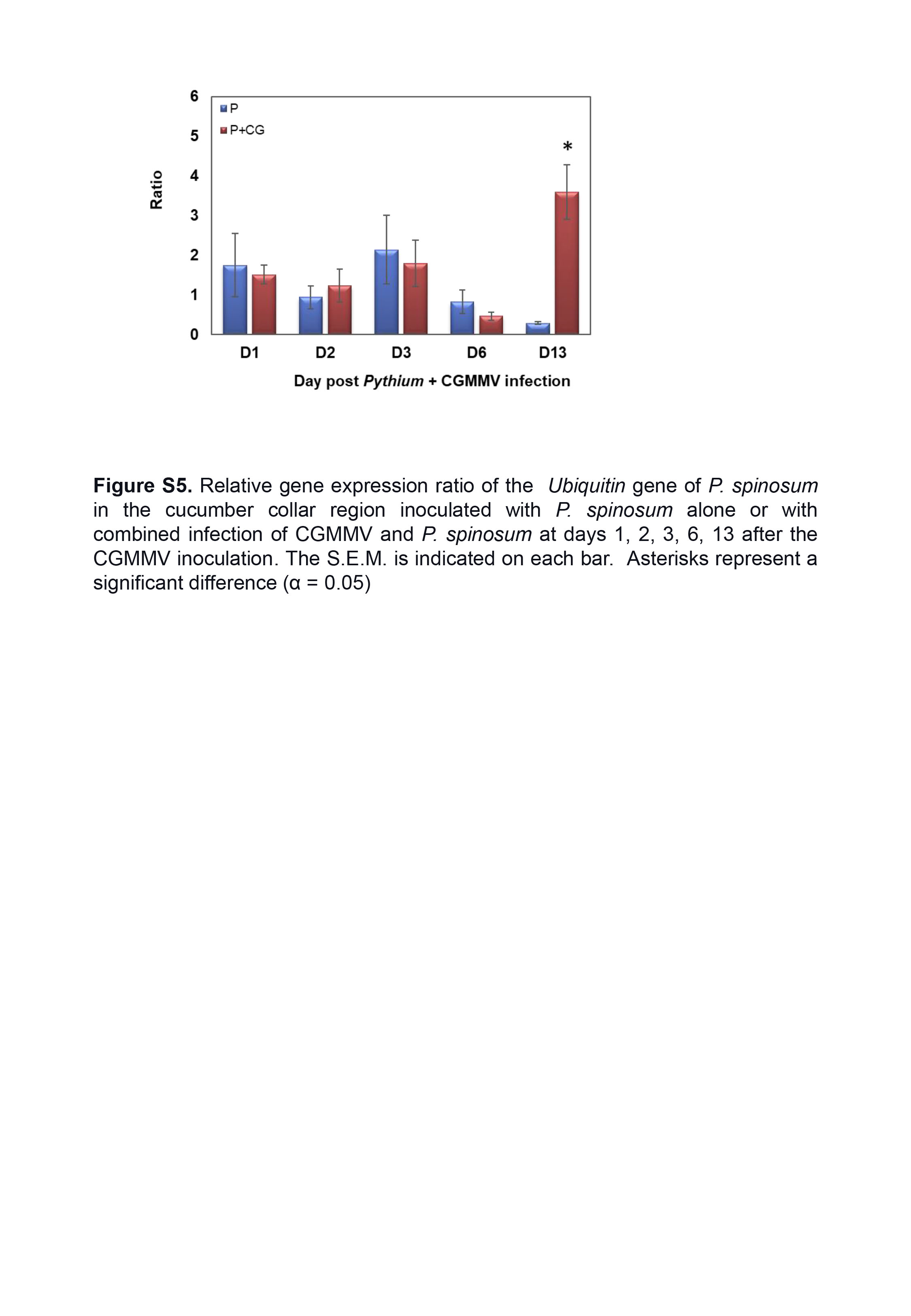

Supplement: Supplementary file 5 [file Image_5.jpeg]
